# Isolation of Highly Active Monoclonal Antibodies against Multiresistant Gram-Positive Bacteria

**DOI:** 10.1371/journal.pone.0118405

**Published:** 2015-02-23

**Authors:** Friederike S. Rossmann, Diana Laverde, Andrea Kropec, Felipe Romero-Saavedra, Melanie Meyer-Buehn, Johannes Huebner

**Affiliations:** 1 Department of Medicine, Division of Infectious Diseases, University Hospital, Freiburg, Germany; 2 Faculty of Biology, Albert-Ludwigs-University, Freiburg, Germany; 3 Department of Pediatrics, Dr. von Hauner Children’s Hospital, Ludwig-Maximilians University, Munich, Germany; 4 German Center of Infection Research (DZIF), Partnersite Munich, Germany; Amphia Ziekenhuis, NETHERLANDS

## Abstract

Multiresistant nosocomial pathogens often cause life-threatening infections that are sometimes untreatable with currently available antibiotics. Staphylococci and enterococci are the predominant Gram-positive species associated with hospital-acquired infections. These infections often lead to extended hospital stay and excess mortality. In this study, a panel of fully human monoclonal antibodies was isolated from a healthy individual by selection of B-cells producing antibodies with high opsonic killing against *E. faecalis* 12030. Variable domains (VH and VL) of these immunoglobulin genes were amplified by PCR and cloned into an eukaryotic expression vector containing the constant domains of a human IgG1 molecule and the human lambda constant domain. These constructs were transfected into CHO cells and culture supernatants were collected and tested by opsonophagocytic assay against *E. faecalis* and *S. aureus* strains (including MRSA). At concentrations of 600 pg/ml, opsonic killing was between 40% and 70% against all strains tested. Monoclonal antibodies were also evaluated in a mouse sepsis model (using *S. aureus* LAC and *E. faecium*), a mouse peritonitis model (using *S. aureus* Newman and LAC) and a rat endocarditis model (using *E. faecalis* 12030) and were shown to provide protection in all models at a concentration of 4 μg/kg per animal. Here we present a method to produce fully human IgG1 monoclonal antibodies that are opsonic *in vitro* and protective *in vivo* against several multiresistant Gram-positive bacteria. The monoclonal antibodies presented in this study are significantly more effective compared to another monoclonal antibody currently in clinical trials.

## Introduction

Infections caused by multiresistant nosocomial pathogens are one of the major problems in modern medicine. A recent report from the Centers for Disease Control and Prevention (CDC) estimates that in the US about two million people acquire serious infections with resistant bacteria, and that probably about 23,000 patients die each year as a direct consequence of these infections. Gram-positive bacteria account for a large proportion of these infections [1], and staphylococci and enterococci are the predominant species associated with these hospital-acquired infections [2].

One of the major problems involves enterococci, mainly *Enterococcus faecium* resistant to vancomycin (VRE) and most of them belonging to the clonal complex 17 (CC17). These bacteria cause bloodstream infections, urinary tract infections and foreign-body infections (e.g. catheters, stents, CNS shunts, artificial heart valves etc.) [3] mostly in immunocompromised patients. For the US it is estimated that about 66,000 enterococcal infections occur each year, and about 20,000 of these are due to multiple-drug-resistant (i.e. VRE) with about 1,300 death per year. High rates are also seen for *Staphylococcus aureus* infections that are resistant to methicillin (MRSA), causing mostly pneumonia, skin-, wound-, bloodstream- and surgical site infections [4]. About 80,000 *S. aureus* infections have been reported in the US per year with about 12,000 deaths caused by bacteria resistant to oxacillin/methicillin [2].

Here we present a discovery platform to identify antibodies from healthy individuals that are protective against multiresistant pathogens and can be used for passive immunotherapy of these infections.

## Materials and Methods

### Ethics statement

All animal experiments were performed in compliance with the German animal protection law (TierSchG). The animals were housed and handled in accordance with good animal practice as defined by FELASA and the national animal welfare body GVSOLAS. The animal welfare committees of the University of Freiburg (Regierungspraesidium Freiburg Az 35/9185.81/G-12/070 and Az 35/9185.81/G-07/72) approved all animal experiments. The institutional review board of the University of Freiburg approved the study protocol. Moribund animals or animals in distress from infection (paucity of movement, ruffeled fur, reduced feeding or drinking) were humanely eutanized by CO2 asphyxation. Animals were watched closely during the course of the experiment (i.e. at least every 4 hours).

Collection of blood from human subjects was approved by the Ethics Committee of the University of Friburg (approval 116/04). Written consent was obtained prior to collection of blood from healthy donors.

### Bacterial Strains and Plasmids

Bacterial strains and plasmids used in the present study are shown in Table 1. *E. coli* were grown with agitation at 37°C in Luria broth (LB; Roth) or LB Agar, while gram-positive bacteria (*S. aureus, E. faecalis* 12030 and *E. faecium*) were grown in Tryptic Soy Broth (TSB) or Tryptic Soy Agar (TSA) at 37°C without agitation. Antibiotics (all purchased from Sigma) were added as indicated.

**Table 1:** Bacterial strains, Antibodies and Plasmids.

Species	Description		Reference
*E. coli* Top 10	Competent cells		Invitrogen
*E. faecalis* 12030	Clinical Isolate		[33,34]
*E. faecium 1162*			[35]
*S. aureus* LAC	CA-MRSA USA400		[10]
**Antibodies**			
α-LTA	rabbit serum raised against purified LTA from *E. faecalis* 12030		[7]
NRS	normal rabbit serum (Cedarlane Labs		
mouse α-LTA mAb	IBT BIOSERVICES, Gaithersburg MD		
VH4E, VH8			this study
*S. aureus* Newman	USA300		[25]
**plasmids**		**Antibiotic resistance**	
TOPO 2.1	Cloning vector	Ampicillin; Kanamycin	Invitrogen
TCAE6.7	Eukaryotic expression vector containing human IgG1 and lambda domain	Ampicillin; Neomycin	[10]

### Opsonophagocytic assay

Opsonophagocytic killing was assessed as described by Theilacker *et al*. [5] using 1.7% baby rabbit serum (Cedarlane) as complement source, and rabbit sera raised against purified lipoteichoic acid (LTA) from *E. faecalis* 12030 as positive control [6–8]. Bacteria were incubated and grown to mid-exponential (OD_650nm_) phase. Equal volumes of bacterial suspension (2.5 x 10^7^ per mL), leukocytes (2.5 x 10^7^ per mL), complement source (1.7% final concentration), and culture supernatant of immortalized and stimulated B-cell cultures or heat-inactivated immune rabbit serum (as control) were combined and incubated on a rotor rack at 37°C for 90 minutes. After incubation, live bacteria were quantified by agar culture of serial dilutions. Percent killing was calculated by comparing the colony counts at 90 min (*t*90) of a control not containing PMNs (PMN^neg^) to the colony counts of a tube that contained all four components of the assay using the following formula: {[(mean CFU PMN^neg^ at *t*90)—(mean CFU at *t*90)]/(mean CFU PMN^neg^ at *t*90)}×100.

### EBV immortalization and identification of opsonic B-cell clones

Blood (10 mL) was taken by venipuncture from healthy volunteers and B-cells were isolated and immortalized as described by Tosato *et al*. [9]. Immortalized cells were cultured in tissue culture plates for 6 days and then stimulated by 40 μg/ml TNP-LPS (Biosearch Technologies), 10 U/mL hIl-1 (BD) and 100 U/ml hIl-2 (BD). The supernatant of each well was collected and used in an opsonophagocytic killing assay (OPA) against *E. faecalis* 12030 to identify the well resulting in the highest killing. B-cells from this well were distributed into a new tissue culture plate. Supernatants were again tested by OPA and the cells of the well leading to the highest killing were distributed into a new plate. After 4 rounds, B-cells in the wells with the strongest response were lyzed and mRNA and cDNA was prepared.

### Amplification of variable domains

Immortalized B-cells were cultured after the final round of selection for about 8 weeks until sufficient numbers for RNA preparations were obtained. RNA was extracted from about 5 × 10^6^ immortalized cells using the RNeasy kit (QIAGEN) according to the manufacturer's instructions. A 500 ng volume of total RNA was reverse-transcribed using the Omniscript kit (QIAGEN) and 1 μl volume of the cDNA product was used as a template for PCRs. Primers for the amplification of the variable regions are listed in Table 2. Each reaction consisted of 50 μl PCR Mix (HotStart Taq DNA Polymerase, QIAGEN), 100 pmol of each primer [10], and 1 μl cDNA template. For PCR amplification 35 cycles were used with the following protocol: 95°C for 30 s initially followed by cycles of 95°C for 30 s, 58°C for 30 s, and 68°C for 45 s, with a final extension at 70°C for 10 min. PCR products were cloned into the TOPO cloning vector 2.1 (Invitrogen) and sequenced. The resultant sequences were compared against known germ line sequences using IgBLAST (http://www.ncbi.nlm.nih.gov/igblast).

**Table 2:** Primers used for the amplification of the variable domains.

light chains	
Lam1	5′AGATCTCTCACCATGGCCRGCTTCCCTCTCCTC
Lam2	5′AGATCTCTCACCATGACCTGCTTCCCTCTCCTC
Lam3	5′AGATCTCTCACCATGGCCTGGGCTCTGCT
Lam4	5′AGATCTCTCACCATGACTTGGATCCCTCTCTTC
Lam5	5′AGATCTCTCACCATGGCATGGATCCCTCTCTTC
Lam6	5′AGATCTCTCACCATGGCCTGGACCCCTCTCTGG
Lam7	5′AGATCTCTCACCATGGCCTGGATGATGCTTCTC
Lamconstant	5′GACCGAGGGGGCAGCCTTGGGCTGACCTAGG
For the light chain primers the Bgl II site is underlined and for the lambda constant primer the Avr II site is underlined.	
**heavy chains**	
VH1	5′GTCGACATGGACTGGACCTGGA
VH2	5′GTCGACATGGACATACTTTGTTCCAC
VH3	5′GTCGACATGGAGYYKGGGCTGAGC
VH4	5′GTCGACATGAACAYCTGTGGTTCTT
VH5	5′GTCGACATGGGGTCAACCGCCATCCT
VH6	5′GTCGACATGTCTGTCTCCTTCCTCAT
VH7	5′GTCGACATGAAACATCTGTGGTTCTTC
Heavyconstant	5′TGGGCCCTTGGTGCTAGCTGAGGAGAC
For the heavy chain primers the Sal I site is underlined and for the heavy constant primer the Nhe I site is underlined.	

### Cloning of variable domains into eukaryotic expression vector TCAE6.7

The TCAE6.7 vector containing the human lambda and IgG1 constant region was used as previously described [11,12]. Heavy (H) chain V-region genes from the four constructs were digested with SalI and NheI restriction enzymes (NEB) and ligated into TCAE6.7 cut with the same enzymes. The ligation reaction mixture was transformed into competent *E. coli* TOP10 cells (Invitrogen) and plasmids were purified using a plasmid Miniprep kit (QIAGEN). The vector was sequenced to confirm the correct sequence. For light (L) chains, variable domains of the light chain cloned into the TOPO cloning vector 2.1 were digested with BglII and AvrII restriction enzymes (NEB) and ligated with the TCAE6.7 vector already containing the matching H chain variable region and cut with the same enzymes. Plasmids were transformed into *E. coli TOP10 cells (Invitrogen)*, individual colonies were isolated, plasmids were obtained, and the inserted DNA was sequenced to ensure that the correct L chain V-region was cloned into the eukaryotic expression vector. Since IgG1 has been reported to be superior to IgG3 in complement-mediated killing of bacteria [13], we used IgG1 constant domains.

### Transfection of CHO cells and expression of the recombinant antibody molecules

Two constructs containing the different H chains (VH4E and VH8, see Table 3) combined with the L chain were created and were transfected separately into Chinese Hamster Ovary (CHO) DHFR^−/−^ cells by using Lipofectamine 2000 (Invitrogen) according to the manufacturer's instructions. Stably transfected cells were selected using medium without nucleotides (Biochrom). Culture supernatants of the transfected CHO cells were harvested daily for 8 days. Supernatants containing monoclonal antibodies were pooled, precipitated with ammonium sulfate (35% w/v), washed and dialyzed against phosphate-buffered saline (PBS) (Biochrom) using Slide-A-Lyzer dialysis cassettes (MWCO 10; Thermo Scientific). Monoclonal antibody (mAb) concentrations were determined by ELISA using the standards and the kit from General Bioscience.

**Table 3:** Sequences of heavy and light chain of the 4 antibodies isolated after the 4th round of selection.

Top V gene match	Top D gene match	Top J gene match	Chain type	Stop codon	V-J frame	Productive
IGHV1–69*06	IGHD3–9*01	IGHJ2*01	VH	no	in-frame	yes
IGHV1–2*02	IGHD1–7*01	IGHJ6*04	VH	no	in-frame	yes
IGLV1–51*01	—	IGV1*01	VL	no	in-frame	yes

Sequences have been analyzed with IgBlast.

### Opsonphagocytic inhibition

*E. faecalis* 12030 was either treated with 1M NaIO_4_ for 24 h in the dark or with Proteinase K (1mg/ml, incubated in a shaking water bath at 54°C for 4 h). After 1M NaIO_4_ treatment, ethylene glycol was used to neutralize excess NaIO_4_. After Proteinase K treatment, extracts were incubated for 1 h at 65° to inactivate Proteinase K and washed 3 times with saline. For inhibition studies, either pre-treated bacterial cells or purified lipoteichoic acid was used as inhibitor. VH4E (120 pg/mL) and VH8 (120 pg/mL) was diluted 1:50 and incubated for 60 min at 4°C with an equal volume of a solution containing 20 or 100 μg cell wall extract (treated either with NaIO_4_ or Proteinase K) from *E. faecalis* 12330 or 100 and 20 μg/mL lipoteichoic acid from *S. faecalis* (*E. faecalis*) purchased from Sigma (St. Louis,Mo.). Subsequently, the respective antibody was used in the OPA as described above. Inhibition assays were performed at serum dilutions yielding 50–80% killing of the inoculum without the addition of the inhibitor. The percentage of inhibition of opsonophagocytic killing was compared to controls without inhibitor [7].

### Measurement of lipoteichoic acid specific IgG titers in monoclonal antibody preparations

The total IgG concentration was determined for each monoclonal antibody preparation, with the Easy-Titer Human IgG Assay kit (Thermo Scientific) according to the manufacturer’s instructions. Specific IgG titers against lipoteichoic acid were measured by ELISA as described previously [14]. In brief, Nunc-immuno Maxisorp MicroWell 96 well plates were coated with 0.125 μg LTA from *S. aureus* purchased from Sigma (St. Louis, Mo.) in 0.2M carbonate-bicarbonate coating buffer. Plates were incubated overnight at 4°C, washed three times after incubation with PBS containing 0.05% Tween 20, and blocked with 3% bovine serum albumin (Applichem GmbH) in PBS at 37°C for 2 hours. Rabbit sera were plated in twofold serial dilutions and incubated 1 hour at 37°C, using three and four fold dilutions of each monoclonal antibody preparation. Alkaline-phosphatase-conjugated anti-human IgG produced in goat (Sigma) diluted 1:1,000 was used as secondary antibody and p-nitrophenyl phosphate (Sigma) was used as substrate (1mg/mL in 0.1M glycine, 1mM MgCl2, 1mM ZnCl2, pH 10.4). After 60min of incubation at room temperature, the absorbance was measured at 405nm on a Tecan Infinite 200 PRO (Tecan Group Ltd.). Each experiment was performed twice at different time-points, and wells were measured in triplicate. The IgG titers were calculated as follows: for each sample, a plot of OD value against the antibody dilution [Log_10_(antibody dilution)] was used to calculate the dilution of monoclonal antibody giving an absorbance of 1 at 405nm after 60min of incubation. The value extrapolated from the standard curve was calculated to generate the final titer [15,16].

### Mouse sepsis model

The protective efficacy of the monoclonal antibodies was tested against *E. faecium* and *S. aureus* LAC in a mouse bacteremia model as described previously [6]. Eight female BALB/c mice 6–8 weeks old (Charles River Laboratories Germany GmbH) were infected by i.v. injection of *E. faecium* (1.8 x 10^8^ cfu) or *S. aureus* (5.0 x 10^7^ cfu) via the tail vein. Antibodies were given 48 h and 24 h prior to bacterial challenge i.p. in 200 μl saline. Mice were sacrificed 48 h (LAC) and 24 h (*E. faecium*) after infection and livers were aseptically removed, weighted and homogenized. Bacterial counts were enumerated by serial dilutions on TSA plates after overnight incubation. Statistical significance was assessed by Mann-Whitney test [7].

### Rat endocarditis model

Female Wistar rats (Charles River Laboratories Germany GmbH), weighing 200 to 300g were used in a previously described rat endocarditis model [17]. Animals were anesthetized by i.m. application of 5.75% ketamine and 0.2% xylazine. Nonbacterial thrombotic endocarditis was caused by insertion of a small plastic catheter (polyethylene tubing; Intramedic PE 10) via the right carotid artery. Inoculation of bacteria was done after 48 h via injection into the tail vein. Rats were challenged with *E. faecalis* 12030 (1.25 x 10^5^ cfu per animal) and 4 animals received the monoclonal antibody VH4E while 4 received normal rabbit serum (NRS). Animals were sacrificed on postoperative day 6 and the correct placement of the catheter was confirmed in 6 rats (3 of each group). Two rats with incorrect catheter placement were excluded from the final analysis. Endocarditis was assessed and graded macroscopically, and valve vegetations were removed, weighted and homogenized. The primary evaluation criterion was the bacterial count in the vegetation (cfu per gram and per ml, respectively). The mean and standard deviation was calculated for each group.

### Protection Studies

Bacterial strains *S. aureus* Newman and LAC (USA 300) were grown overnight in TSB. Cells were harvested by centrifugation (4000 r.p.m., 30min, 4°C), washed and diluted to the correct concentration in 0,9% NaCl to obtain a bacterial inoculum of 2x10^9^ cfu/mL (inoculum stocks are stored at -80°C). Eight female Balb-C mice 5–6 weeks old (Charles River Laboratories Germany GmbH) were passively immunized by intraperitoneal injection of 200μL of either monoclonal antibody VH8 (diluted 1:50 in 0.9%NaCl corresponding to 4 ug/kg) or normal rabbit serum (NRS) 24 hours before challenge. Mice were infected by intraperitoneal injection of 200μL of the bacterial inoculum (*S. aureus* Newman). The amount of bacteria in the inoculum was verified by serial dilution and plating. Infected animals were monitored for morbidity or recovery over a period of 72 hours. For the protection studies comparing VH8 and the mouse anti-lipoteichoic monoclonal antibody, six female Balb-C mice 5–6 weeks old (Charles River Laboratories Germany GmbH) were passively immunized by intraperitoneal injection of 200μL of the monoclonal antibodies 24 hours before bacterial challenge with *S. aureus* LAC. Monoclonal antibody VH8 was adjusted to a final concentration of 12 ng/mL, while mouse anti-lipoteichoic monoclonal antibody (IBT BIOSERVICES) was applied at 3000 ng/mL or 12 ng/mL in 0.9% NaCl. Normal rabbit serum (NRS) and rabbit polyclonal anti-lipoteichoic acid serum [7] were used as negative and positive controls, respectively. Mice were infected by intraperitoneal injection of 200μL of bacterial inoculum. Infected animals were monitored for morbidity or recovery over a period of 24 hours.

### Statistical Analysis

All statistical testing was done using GraphPad PRISM with ANOVA and Dunnett, or Tukey post tests as indicated. Two-group comparisons were done by unpaired t-test.

## Results

**Fig 1:**
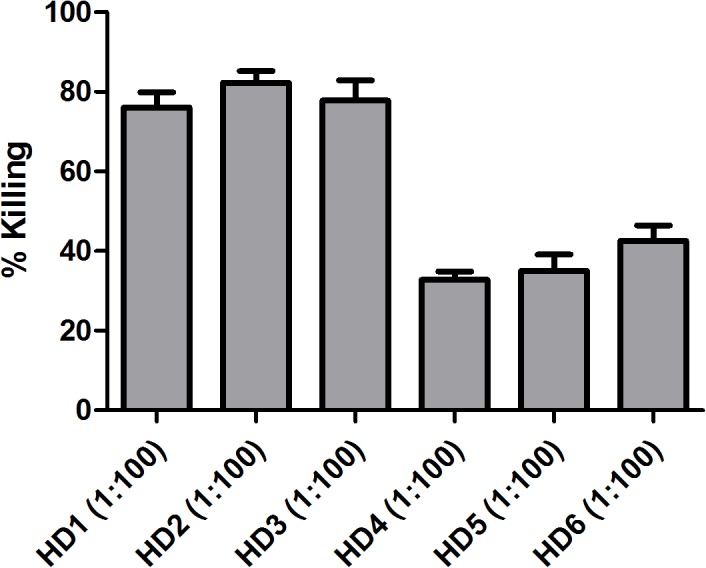
Opsonic killing of sera from healthy human volunteers. Opsonophagocytic killing against *E. faecalis* 12030 was assessed in sera at a dilution of 1:100. Statistical analysis was done by ANOVA with Dunnett post test. * p<0.05, ** p<0.01, *** p<0.001. Error bars represent standard error of the mean.

A pre-screen of a donor pool by opsonophagocytic assay (OPA) was used to identify the donor with the highest titers of opsonic antibodies against *E. faecalis* 12030. Healthy donor 2 showed the highest opsonic killing (82%) using 1:100 serum (Fig. 1).

B-cells of donor 2 were immortalized using EBV, spread into tissue culture plates, and undiluted supernatants were tested by opsonophagocytic assay against *E. faecalis* 12030. The well with the highest opsonic killing was selected, and B-cells in the respective well were removed, cultured, and subsequently seeded into a new tissue-culture plate. After the 4th round, the content of the well with the highest titer was used to prepare mRNA and cDNA, and sequencing revealed the presence of one light chain variable domain, and 2 different heavy chain variable domains (see Table 3). After cloning of these heavy-light chain pairs into TCAE and transfection of these constructs into CHO cells, the recombinant monoclonal antibodies from the supernatants were used to study the target of the monoclonal antibodies.

**Fig 2:**
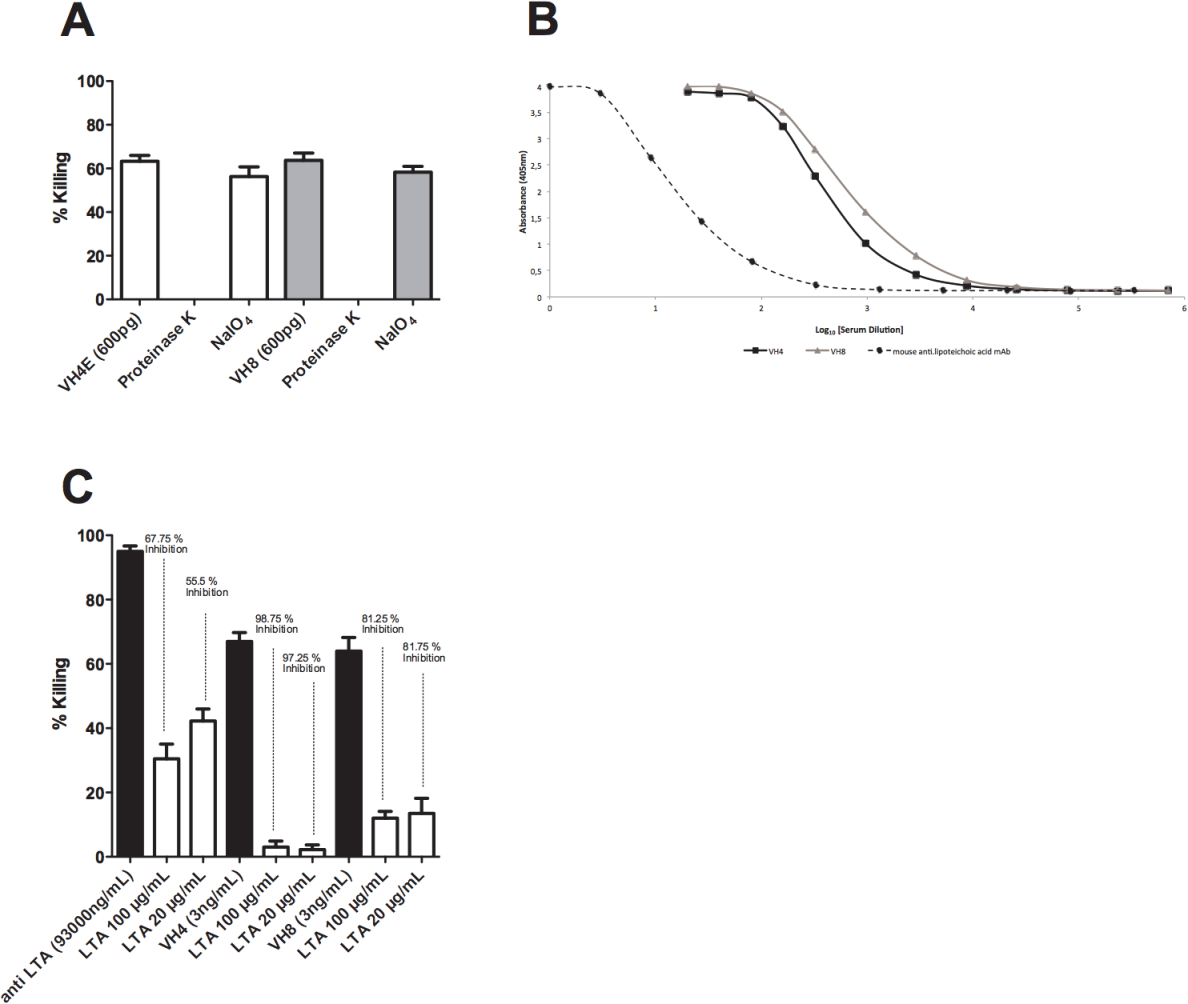
Determination of the target of VH4E and VH8. (A) Opsonophagocytic Inhibition Assay after absorbing mAbs with *E. faecalis* 12030 treated with Proteinase K or NaIO_4_ (B) Binding of VH4E and VH8 to LTA is shown in an ELISA coating wells with *S. aureus* LTA (C) Osonophagocytic Inhibition Assay absorbing monoclonal antibodies with LTA from *S. aureus*. **(A)** Killing of VH4E and VH8 against *E. faecalis* 12030 is about 65% (first white or grey column) and killing is completely abolished when bacteria are treated with proteinase K before absorption. Opsonic activity of VH4E and VH8 is not inhibited when mAbs are absorbed with *E. faecalis* treated with NaIO_4_ indicating that a polysaccharide is the target of the mAbs. **(B)** Binding of VH8 and the mouse anti-lipoteichoic monoclonal antibody to LTA was tested in an ELISA. As coating antigen LTA from *S. aureus* was used and serum dilutions are indicated in the X axis. Each point represents the average of two measurements. **(C)** An Opsonophagytosis assay after absorbing monoclonal antibodies with purified LTA confirmed the results of the ELISA. Opsonophagocytic killing was compared to controls from which leukocytes were obtained. Statistical analysis was done by ANOVA with Dunnett post test. * p<0.05, ** p<0.01, *** p<0.001. Error bars represent standard error of the mean.

An opsonophagocytic Inhibition Assay (OPIA) was performed with mAbs VH4E and VH8 showing the highest killing against the tested strains to determine their bacterial target. Cell wall extracts of *E. faecalis* 12030 were treated with Proteinase K or NaIO_4_ to assess if a polysaccharide or a protein antigen is the target of the mAbs. Opsonic activity of VH4E and VH8 was not inhibited when absorbing bacteria were treated with NaIO_4_, but was inhibited when bacteria were treated with proteinase, indicating that a polysaccharide is the target of the mAbs (Fig. 2A).

The binding affinity of the monoclonal antibodies was tested in an ELISA to assess whether VH8 is directed against LTA, a carbohydrate-containg surface-exposed antigen. Binding of VH4E and VH8 against staphylococcal LTA was compared to a mouse anti-lipoteichoic monoclonal antibody from *S. aureus*, known to be directed against LTA. Affinity of VH8 and VH4E against LTA was clearly higher compared to the mouse anti-lipoteichoic monoclonal antibody (Fig. 2B).

Using LTA as an inhibitor in an opsonophagocytic inhibition assay, we confirmed the specificity of VH8 and VH4E since concentrations of 100 μg/mL effectively inhibited killing by 67.75% and 98.75%, respectively (Fig. 2C).

**Fig 3:**
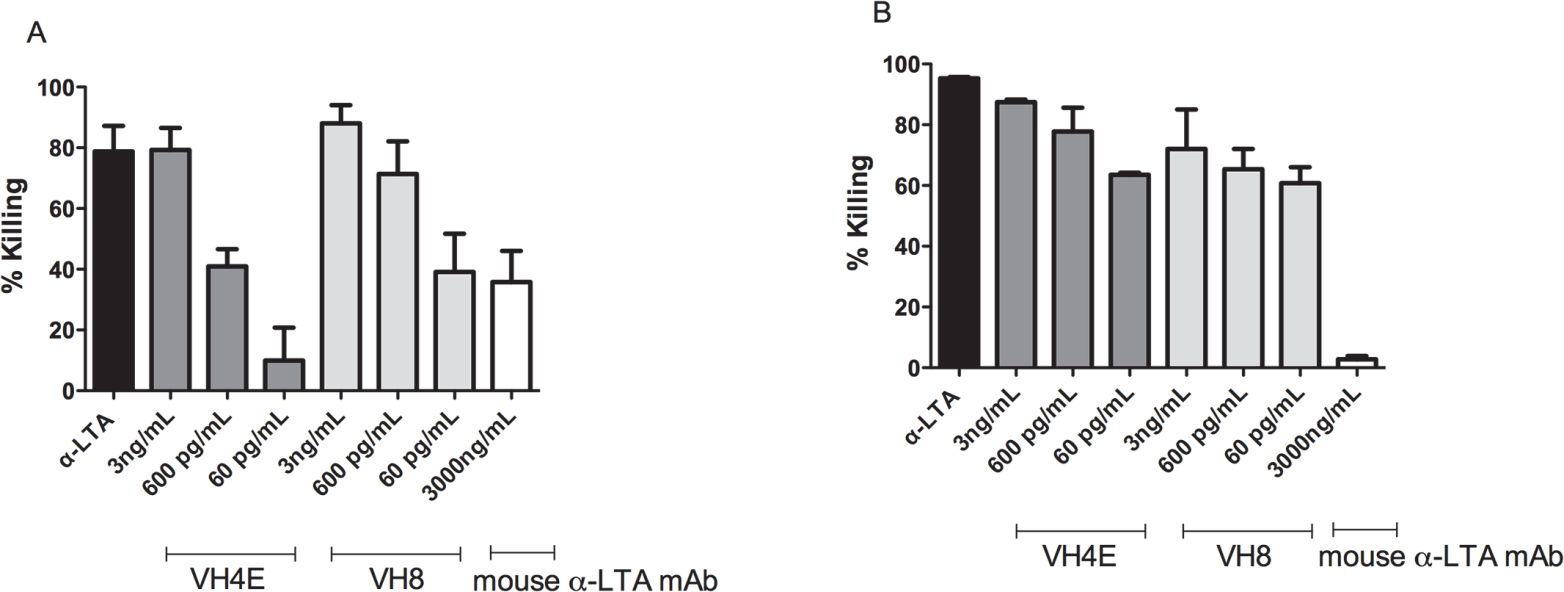
Measurement of opsonophagocytic killing of monoclonal antibodies VH4E and VH8 and the mouse anti-lipoteichoic monoclonal antibody against (A) MRSA strain LAC and (B) *E. faecalis* 12030. The opsonophagocytic assay was performed using baby rabbit serum as complement source and rabbit sera raised against purified LTA from *E. faecalis* 12030. VH4E and VH8 show high opsonic killing against both strains in a dose dependent manner and are clearly more efficient than the mouse anti-lipoteichoic monoclonal antibody, which showed modest killing against LAC at very high concentrations (i.e. 1,000x). Opsonophagocytic killing activity was compared to controls from which leukocytes were omitted. Statistical analysis was done by ANOVA with Dunnett post test. * p<0.05, ** p<0.01, *** p<0.001. Error bars represent standard error of the mean.

In an opsonophagocytic assay, killing was tested against *E. faecalis* 12030 and *S. aureus* LAC (CA-MRSA). Opsonic killing occurred in the presence of monoclonal antibodies in a dose-dependent manner (79–88% at the highest concentration of 3ng/mL against both strains), whereas the absence of the mAbs but presence of neutrophils and complement alone did not reduce viable counts (Fig. 3). A lipoteichoic acid-specific serum (αLTA T5) was used as positive control because we have shown previously that this serum is opsonic and protective against enterococcal strains [7].

**Fig 4:**
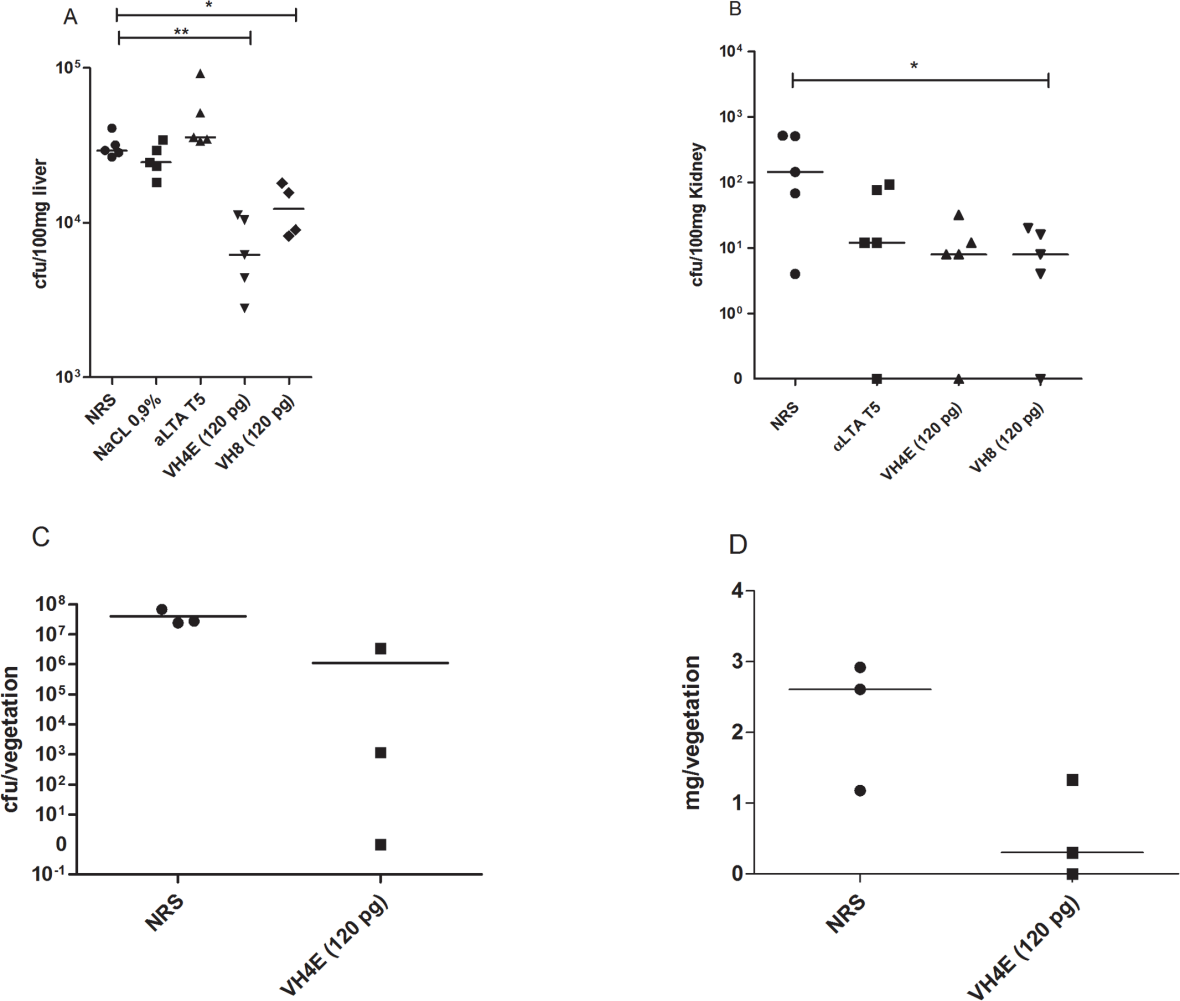
Investigating efficacy of the monoclonal antibodies in two independent animal models. A mouse bacterial sepsis model was tested for the MRSA strain LAC (A) and *E. faecium* (B). A rat endocarditis model was confirms the protection of VH4E (C and D). In the mouse bacteremia model *E. faecium* (1.8 x 10^8^ cfu) or *S. aureus* (5.0 x 10^7^ cfu) was injected i.v. into the tail vein. Cfu in the liver and the kidney was assessed after 24h. Statistical analysis was done by ANOVA with Tukey post test (a and b). * p<0.05, ** p<0.01, *** p<0.001. Error bars represent standard error of the mean. A rat endocarditis model confirmed protection against *E. faecalis* 12030 by VH4E (at a total concentration of 10 pg). Inoculation of bacteria was done 48 h after catheter placement via injection into the tail vein. Bacterial counts in the vegetations (cfu per mg vegetation) are shown in (C), and (D) shows the absolute weights of explanted vegetation. Differences in mg per vegetations are significant (p < 0.05) and were tested by unparied t-test (c and d). * p<0.05, ** p<0.01, *** p<0.001. Error bars represent standard error of the mean.

Passive immunotherapy with monoclonal antibodies VH4E and VH8 was studied in a mouse bacteremia model. In this model we could demonstrate that VH4E and VH8 promote clearance of *E. faecium* E1162 and *S. aureus* LAC, whereas normal rabbit sera (NRS) did not protect from bacterial infection. The number of bacteria recovered from the liver and kidney of mice infected with both strains was significantly reduced compared to those not being treated with the mAbs (Fig. 4A and 4B). A lipoteichoic acid-specific serum (αLTA T5) was used as positive control.

Comparing monoclonal antibody VH4E with normal rabbit serum (NRS) in a rat endocarditis model, bacterial vegetations of VH4E-treated rats were significantly reduced (measured in cfu per milliliter and in milligram vegetation), compared to those not being treated with VH4E the day before bacterial challenge (Fig. 4C and 4D).

**Fig 5:**
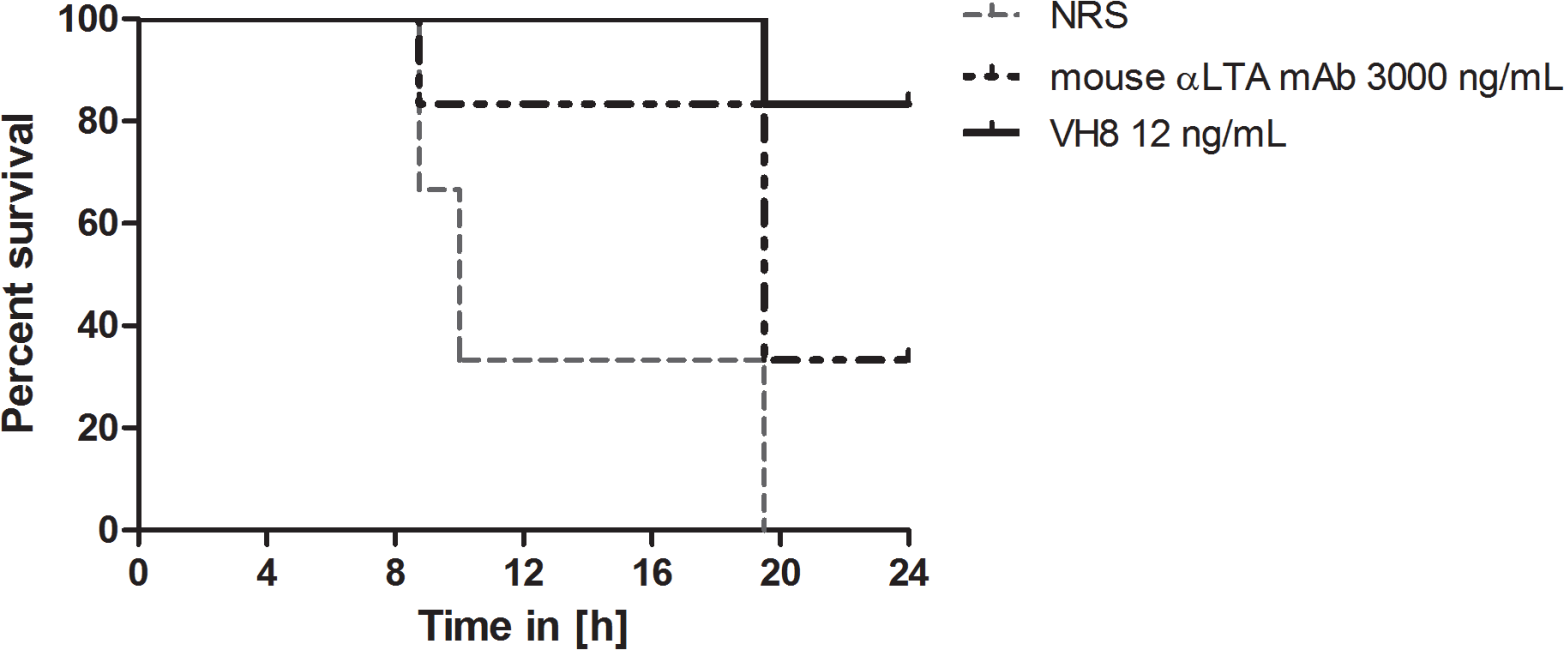
Protection against *S. aureus* LAC infection with VH8 and mouse anti-lipoteichoic monoclonal antibody. A dose of 12ng/mL VH8, 3000ng/mL mouse anti-lipoteichoic monoclonal antibody and 200 μl of Normal Rabbit Serum were given to animals 24 hours before bacterial challenge. Strain *S. aureus* LAC was used at a challenge dose of 2x10^9^ cfu/mouse (6 mice per group). Protection by different sera was observed for VH8 (5/6 mice survived) while the mouse anti-lipoteichoic monoclonal antibody and the NRS groupdid not result in protection.

In a different animal model, *S. aureus* Newman was injected i.p. and mice received VH8 (4 μg/kg per mouse in 200 μl saline) 24 hours before bacterial challenge. At an inoculum of 2 x 10^9^ per mouse, all mice receiving NRS died after 18 hours, while 3/8 (37.5%) of animals receiving the monoclonal antibody survived (S1 Fig.). To compare the efficacy of VH8 and the mouse anti-lipoteichoic monoclonal antibody, we passively immunized mice with VH8, the mouse anti-lipoteichoic monoclonal antibody, normal rabbit serum, and anti-LTA serum 24 hours before bacterial challenge with *S. aureus* LAC. As shown in Fig. 5, only 1/6 mice (83%) receiving VH8 died during the observation period of 19.5 hours, while 4/6 mice (33%) died when 3 mg/ml (a 250x higher concentration) of the mouse anti-lipoteichoic monoclonal antibodywas given. In the control group treated with normal rabbit serum, all animals died and when the mouse anti-lipoteichoic monoclonal antibody was given at the same concentration as for VH8 (12 ng/mL), all mice died after 8.75 hours (S1 Table). In this experiment, an anti-LTA serum that was raised against LTA form *E. faecalis* 12030 did also not protect animals (1/6 mice survived).

## Discussion

Research and development of new antibiotics has declined dramatically or has been completely abandoned by most large pharmaceutical companies, mostly because of reduced profit margins [18]. On the other hand, the more than 40-year history of clinical trials of anti-inflammatory strategies for the treatment of sepsis was described as 'graveyard' for the pharmaceutical industry, especially regarding Gram-negative bacteria [19]. Several reasons for this assessment have been mentioned: a) unsuitable animal models, b) inhomogeneity of the patient population enrolled in clinical studies, c) definition of sepsis itself (which is complicated because of the different underlying conditions and dynamic time courses) [20]. However, the rapid rise of multidrug-resistant pathogens, as demonstrated by the recent report from the Centers for Diseases Control [2], illustrates the seriousness of the situation and classifies frighteningly many pathogens (e.g. VRE, *Pseudomonas aeruginosa*, MRSA) as especially dangerous, since these are often difficult or sometimes impossible to treat with currently available antibiotics [18].

In contrast to antibiotics, antibodies can be given prophylactically and could protect patients between one and four weeks from hospital-acquired infections [21]. Application of a preformed monoclonal antibody would protect patients immediately, while active immunization requires often booster dosages because the host needs time to develop and produce its own protective antibodies [22,23]. The use of monoclonal antibodies (mAbs) instead of antibiotics would be advantageous also because it enables prophylaxis of patients at particular risk, such as patients in intensive care units, patients after organ or bone-marrow transplantation, patients after implantation of foreign materials (e.g. artificial heart valves or artificial joints), or after chemotherapy. Development of resistance or immune evasion is very unlikely if highly conserved structures (such as capsules or other cell-wall carbohydrate antigens) are targeted [24]. For the development of mAbs it would be advantageous to choose variable domains that recognize cross-reactive antigens to cover a broad spectrum of pathogens [10,25].

Here we describe two human monoclonal antibodies that bind polysaccharide structures of different Gram-positive bacteria and these bacteria can be subsequently eliminated by antibody-mediated complement deposition and phagocytosis (opsonizing antibodies). The strong opsonizing activity could be demonstrated against two different enterococcal and two different staphylococcal strains, among these a multidrug-resistant *S. aureus* (MRSA) and an *E. faecium*. Three different animal models showed that the administration of the monoclonal antibodies protected against infection also *in vivo*. However, the proposed treatment and/or prophylaxis with a monoclonal antibody will probably complement rather than replace current treatment regimens and may act synergistically with antibiotic therapy.

The selection process of the antibodies described was carried out based on functions (i.e., uptake and killing of pathogens by phagocytes) and not—as in most approaches—on affinity. We used as starting material the variety of antibodies in healthy individuals under the assumption that healthy people have preformed protective mechanisms that eliminate a variety of common pathogens. These "natural antibodies" are produced by specific B-cells and are directed against common microbial patterns, which are often polyreactive against widespread bacterial structures and may also target several pathogenic species. The method described shows a "proof of principle" which can be extended to a number of relevant hospital pathogens.

Our results indicate that the antibodies presented here are directed against lipoteichoic acid, an antigen present in most Gram-positive bacteria. We have shown previously that antibodies against LTA can be opsonic, protective, and cross-reactive between different Gram-positive species [7]. Reichmann et al. recently reported that LTA is not surface exposed in *S. aureus* [26]. However, data from other groups [27–29] and from our previous work [7] suggest that some LTA epitopes are exposed on the surface of gram-positive cocci. This may depend on the thickness of the cell wall or capsule (which may be strain-specific) and also on the target of the anti-LTA antibodies used.

In our experiments here, anti-LTA serum did not show significant reduction in colony counts when mice were infected with *S. aureus* LAC. However, in our previous study we did not test *S. aureus* LAC. The reason for the resistance of LAC against anti-LTA serum is unclear at this point and will be explored further. Nonetheless, as shown in Fig. 4A, and in Fig. 5, our monoclonal antibodies were effective against this strain.

A chimeric monoclonal antibody against LTA (pagibaximab) has been previously developed and tested in vitro and in vivo [30–32] by Biosnyexus (Gaithersburg, MD). This antibody consists of the variable domains of a mouse monoclonal antibody and the constant domains of a human IgG. This antibody promoted phagocytosis of staphylococci and inhibited LTA-mediated cytokine induction *in vitro*. At concentrations of 500 μg/ml it improved survival of suckling rats challenged with *S. aureus* and coagulase-negative staphylococci [31]. A randomized, double-blind placebo-controlled phase 2 study was conducted including 88 high-risk neonates with birth-weights between 700 and 1,300 g in 10 neonatal intensive care units (NICUs). The antibody was well tolerated and no staphylococcal or other gram-positive sepsis occurred in neonates treated with pagibaximab [32]. A phase 2b/3 trial "Safety and Efficacy of Pagibaximab Injection in Very Low Birth Weight Neonates for Prevention of Staphylococcal Sepsis" (NCT00646399) was started in 2009 and completed in 2011 including 1,579 neonates, 792 receiving 6 doses of pagibaximab (100 mg/kg) and 787 receiving placebo (i.e. PBS). No publication or official statement regarding the results of this study exist, but information available at ClinicalTrials.gov indicate that 63/792 (7.9%) neonates died in the pagibaximab group while only 53/787 (6.7%) of neonates in the control group died. Regarding the primary endpoint (i.e. cases of staphylococcal sepsis within 35 study days) there were 85/792 (10.2%) cases in the pagibaximab group versus 79/787 (10.0%) in the control group.

Our results differ in several aspects from the data on pagibaximab because the opsonic and protective efficacy of our antibodies is significantly better: in opsonophagocytosis 500 μg/ml pagibaximab [31] vs. 3 ng/ml VH4E and VH8; and in animal models 80 mg/kg pagibaximab [31] vs. 4 μg/kg VH4E and VH8. A direct comparison of the parent pagibaximab mouse monoclonal antibody with our antibodies in the same experiment confirmed these differences regarding affinity, opsonic killing, and protective efficacy in a mouse peritonitis model. In addition, our monoclonal antibodies are fully human compared to the chimeric antibody from Biosynexus, making an anaphylactic or human-anti-mouse-antibody (HAMA/HACA) response unlikely.

The reason for observed difference between pagibaximab and our antibodies may be the exact epitope recognized by the monoclonal antibodies on the relatively complex LTA molecule. We have shown previously that efficient and cross-reactive antibodies are directed against the conserved polyglycerol-phosphate chain of LTA [7]. Sera raised against this epitope are opsonic against *S. aureus, S. epidermidis*, and enterococci [7]. However, using the pagibaximab monoclonal antibody, we did not see efficient killing against enterococci, indicating that this antibody may be directed against a different epitope on the LTA molecule (e.g. the glycolipid anchor, or one of the decorations of the polyglycerol-phosphate chain, such as alanine). While the above-mentioned issues may explain the different efficacy of pagibaximab and our monoclonal antibodies, the results of the phase 2b/3 trial may not represent the clinical efficacy of antibodies against LTA since neonates, and especially premature babies, do not represent a good study cohort, because of their immature immune system and often false-negative blood cultures because of the small volume usually drawn.

While the data presented here are promising, additional *in vitro* studies and better clinical trials are needed to establish monoclonal antibodies against LTA as therapeutic and/or prophylactic strategies against multiresistant gram-positive pathogens.

## Supporting Information

S1 Table Protection against *S. aureus* LAC infection with VH8 and IgG1 mAbs raised against Lipoteichoi acid. (DOCX)

S1 Fig Protection against *S. aureus* Newman infection with VH8 compared to normal rabbit serum. The graph shows percentage of survival during a time period of 72 hours.
(DOCX)
