# Supplementary material for: Isolation of Highly Active Monoclonal Antibodies against Multiresistant Gram-Positive Bacteria
Source: PLoS One. 2015 Feb 23;10(2):e0118405. doi: 10.1371/journal.pone.0118405 (PMC4338075; doi:10.1371/journal.pone.0118405)
Supplement: S1 Table — (DOCX) [file pone.0118405.s001.docx]

|  |  | **Percentage of Survival** | | | | |
| --- | --- | --- | --- | --- | --- | --- |
| **Time after**  **Challenge**  **(in hours)** | **mice** | **NRS** | **Biosynexus**  **3000 ng/mL** | **Biosynexus**  **12 ng/mL** | **VH8**  **12 ng/mL** | **anti-LTA** |
| **1** |  | 100 (6/6) | 100 (6/6) | 100 (6/6) | 100 (6/6) | 100 (6/6) |
| **3** |  | 100 (6/6) | 100 (6/6) | 100 (6/6) | 100 (6/6) | 100 (6/6) |
| **4.5** |  | 100 (6/6) | 100 (6/6) | 100 (6/6) | 100 (6/6) | 100 (6/6) |
| **8.75** |  | 67(4/6) | 83 (5/6) | 0 (0/6) | 100 (6/6) | 17 (1/6) |
| **10** |  | 33 (2/6) | 83 (5/6) | 0 (0/6) | 100 (6/6) | 17 (1/6) |
| **19.5** |  | 0 (0/6) | 33 (2/6) | 0 (0/6) | 83 (5/6) | 17 (1/6) |
| **22** |  | 0 (0/6) | 33 (2/6) | 0 (0/6) | 83 (5/6) | 17 (1/6) |
| **24** |  | 0 (0/6) | 33 (2/6) | 0 (0/6) | 83 (5/6) | 17 (1/6) |

**Table S1: Protection against *S. aureus* LAC infection with VH8 and IgG1 mAbs raised against Lipoteichoic acid.** For the protection studies comparing VH8 and the mouse anti-lipoteichoic monoclonal antibody, six female Balb-C mice 5-6 weeks old were passively immunized by intraperitoneal injection of 200μL of the monoclonal antibodies 24 hours before bacterial challenge with *S. aureus* LAC. Monoclonal antibody VH8 was adjusted to a final concentration of 12 ng/mL, while mouse anti-lipoteichoic monoclonal antibody (IBT BIOSERVICES) was applied at 3000 ng/mL or 12 ng/mL in 0.9 % NaCl. Normal rabbit serum (NRS) and rabbit polyclonal anti-lipoteichoic acid serum (Ref. Theilacker JID 2012) were used as negative and positive controls1/6 mice (83%) receiving VH8 died during the observation period of 19.5 hours, while 4/6 mice (33%) died when 3 ug/ml (a 250x higher concentration) of the mouse anti-lipoteichoic monoclonal antibodywas given. In the control group treated with normal rabbit serum, all animals died and when the mouse anti-lipoteichoic monoclonal antibody was given at the same concentration as for VH8 (12 ng/mL), all mice died after 8.75 hours.
