# Supplementary material for: Isolation of Highly Active Monoclonal Antibodies against Multiresistant Gram-Positive Bacteria
Source: PLoS One. 2015 Feb 23;10(2):e0118405. doi: 10.1371/journal.pone.0118405 (PMC4338075; doi:10.1371/journal.pone.0118405)
Supplement: S1 Fig — The graph shows percentage of survival during a time period of 72 hours. (DOCX) [file pone.0118405.s002.docx]

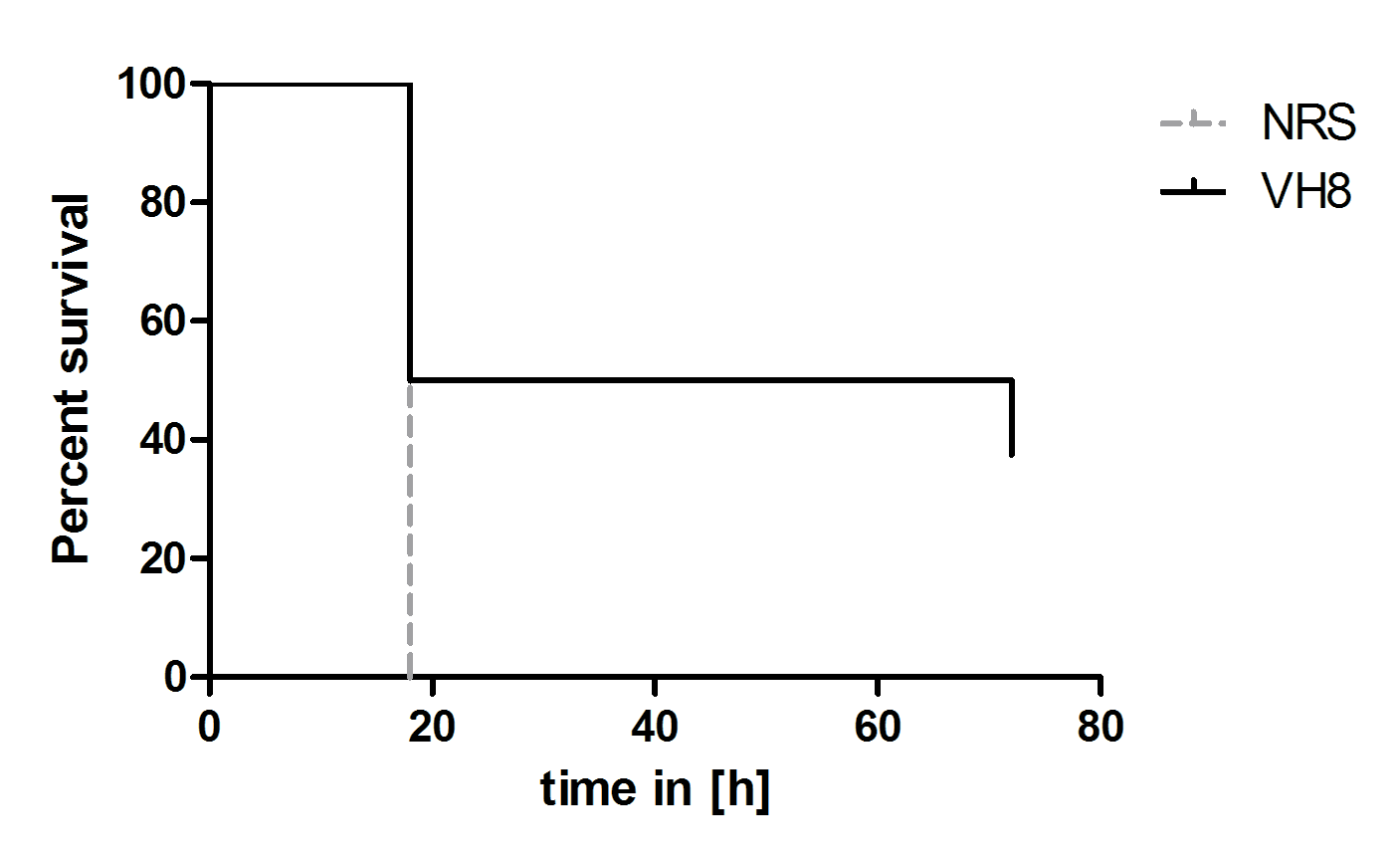


**Figure S1: Protection against *S. aureus* Newman infection with VH8 compared to normal rabbit serum (NRS) (10 mice per group).** A dose of 4 µg/kg of mAb VH8 and 200 µl of Normal Rabbit Serum (NRS) as a control were administered 24 hours before bacterial challenge. Strain Newman was used at a challenge dose of 2 x 10^8^ cfu/mouse. The amount of bacteria in the inoculum was verified by serial dilution and plating. Infected animals were monitored for morbidity or recovery over a period of 72 hours.
